# Reforming support systems of newly diagnosed brain cancer patients: a systematic review

**DOI:** 10.1007/s11060-021-03895-4

**Published:** 2021-11-26

**Authors:** Maria Loizidou, Viktoria Sefcikova, Justyna O. Ekert, Matan Bone, George Samandouras

**Affiliations:** 1 UCL Queen Square Institute of Neurology, University College London, Queen Square, London, WC1N 3BG UK; 2 Wellcome Centre for Human Neuroimaging, University College London, London, UK; 3 Faculty of Biology, Medicine and Health, The University of Manchester, Oxford Road, Manchester, UK; 4 Victor Horsley Department of Neurosurgery, The National Hospital for Neurology and Neurosurgery, Queen Square, London, UK

**Keywords:** Diagnosis, Primary malignant brain neoplasms, Primary malignant brain tumours, Psychosocial support systems

## Abstract

**Purpose:**

Despite the increasing incidence of currently incurable brain cancer, limited resources are placed in patients’ support systems, with reactive utilisation late in the disease course, when physical and psychological symptoms have peaked. Based on patient-derived data and emphasis on service improvement, this review investigated the structure and efficacy of the support methods of newly diagnosed brain cancer patients in healthcare systems.

**Methods:**

This systematic review was performed following the Preferred Reporting Items for Systematic Reviews and Meta-Analysis Protocols. Articles from PubMed, Embase, and CENTRAL databases were screened with six pre-established eligibility criteria, including assessment within 6 months from diagnosis of a primary malignant brain tumour. Risk of bias was evaluated using the Newcastle–Ottawa Scale and Critical Appraisal Skills Program (CASP) Qualitative Studies Checklist.

**Results:**

Of 5057 original articles, 14 were eligible for qualitative synthesis. Four studies were cross-sectional and ten were descriptive. Information given to patients was evaluated in seven studies, communication with patients in nine, and patient participation in treatment decisions in eight. Risk of bias was low in ten studies, moderate in two, and high in two.

**Conclusions:**

Techniques promoting individualised care increased perceived support, despite poor patient-physician communication and complexity of the healthcare system. Extracted data across 14 included studies informed a set of guidelines and a four-step framework. These can help evaluate and reform healthcare services to better accommodate the supportive needs of this patient group.

**Supplementary Information:**

The online version contains supplementary material available at 10.1007/s11060-021-03895-4.

## Introduction

Psychosocial support is a critical but occasionally overlooked domain for cancer patients, particularly at initial disease stages, with resources focusing primarily on diagnosis and treatment [1, 2]. The limited survival and impairment of motor and cognitive abilities in brain cancer makes psychosocial support fundamental [3–5]. Incidence of brain cancer has increased by as much as 39% in the last 30 years [6], leaving more patients to cope with a life-limiting diagnosis without comprehensive support.

Psychosocial needs are important throughout the disease course, yet brain cancer patients tend to seek support after the accumulation of neuropsychological and physical symptoms [7, 8] and after their psychological states become compromised [9–11]. Consequently, without any organised support, patients succumb to ineffective coping mechanisms and reduced compliance to treatment [12, 13]. The current underuse of support services, despite the aforementioned morbidities [14, 15], implies an underlying deficiency in healthcare systems requiring urgent attention. Implementing evidence-based strategies in healthcare systems can maximise the utilisation and efficacy of support services, reduce psychosocial morbidities, and potentially improve prognosis [14, 16].

Support for cancer patients is available at two levels: (i) the macro-level, encompassing support offered by healthcare organisations, and (ii) the micro-level, referring to clinician-patient interactions. At the macro-level, support can be provided in the form of written information about the condition, opportunities for patient involvement in important decisions, and techniques to facilitate patient-healthcare system communication [17–19]. Although engagement of patients in treatment decisions may occur during interactions with their physicians, specific methods for patient  involvement are usually outlined in institution-specific guidelines [20]. At the micro-level, personalised information is provided, including external support services (e.g., counselling) [17, 18, 21]. The interrelation of the two levels is influenced by the treating physicians’ communication style [22]. Although official guidelines guide physician behaviour [23], application of these guidelines is subject to differences in interpersonal skills, information content, and delivery, which impacts patients’ adjustment to their diagnosis [22, 24, 25].

Research has focused on advanced disease stages [26–28], caregivers [28], palliative care [26, 28], and non-medical interventions [28]. The current systematic review is unique in its focus on the psychosocial needs of brain cancer patients at the diagnostic stage, analysing strategies to improve support during this period on the macro- and micro-levels. Based on the available evidence, a list of areas of strength and those requiring improvement has been extracted and analysed.

## Methods

This systematic review has been conducted following the Preferred Reporting Items for Systematic Reviews and Meta-Analysis Protocols (PRISMA-P) [29, 30].

### Eligibility criteria

**Fig. 1:**
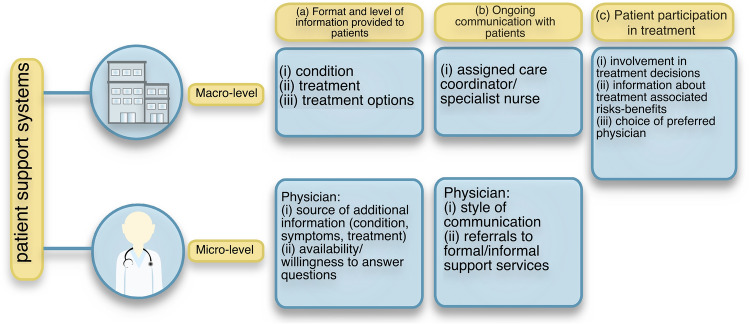
Operational definition of patient support systems. Support is defined as the care offered by healthcare professionals (micro-level) and by the healthcare system in its entirety and/or in accordance to specific guidelines with consistency within institutions (macro-level) in three distinct domains, as perceived by patients: (a) format and level of information provided to patients, (b) ongoing communication with patients, (c) patient participation in treatment

Six inclusion criteria were considered: (i) study type, including randomized controlled trials, cohort studies, case–control studies, cross-sectional studies and qualitative studies, (ii) primary diagnosis of malignant brain tumour, (iii) evaluation within 6 months of diagnosis or if retrospective, referring to the diagnostic period, (iv) adult users of healthcare services (≥ 16 years old), (v) studies relating to support as defined by the operational definition (Fig. 1), and (vi) studies published in peer-reviewed journals, in the English language. Case reports and cohorts of purely metastatic malignant brain tumours were excluded. Studies with mixed cohorts of malignant and non-malignant brain tumours were included if the former constituted the majority or if separate analyses were conducted. Patient and caregiver cohorts were included if extraction of patient data was possible.

### Operational definition

The following were considered in the operational definition for support: (1) NICE guidelines (Quality Standard 15; quality statements 2, 4, 6) [20, 23], (2) research literature [17–19, 21, 22, 24], and (3) multidisciplinary input. Support was defined as the care offered by healthcare professionals (micro-level) and by the healthcare system in its entirety and/or in accordance with specific guidelines (macro-level) consistent within institutions in the three distinct domains, as perceived by patients: (a) format and level of information provided to patients, (b) ongoing communication with patients, (c) patient participation in treatment.

### Search strategy

PubMed, Embase via Ovid, and CENTRAL databases were searched for eligible articles. The review period ranged from November 10, 2020 to December 12, 2020 (see Supplementary data). Reference lists of included articles were manually searched to identify additional studies.

### Study records data management

Literature search results were imported to EndNote (Clarivate, Version X9) for deduplication. Findings were exported to a Microsoft Excel (Microsoft Office, Version 16.16.27) spreadsheet for article screening and selection.

### Selection process

Articles were screened by title and/or abstract by author ML based on the eligibility criteria. Potentially relevant articles, and articles with ambiguous titles and unavailable abstracts, were retrieved full text. Articles chosen for qualitative synthesis were additionally screened by a second author (MB). Any disagreement regarding the eligibility of articles was resolved with consensus.

### Data items and outcomes

Extracted citations from all databases were combined on an Excel spreadsheet, with the following order: author(s), publication year, title, type of publication, language, and abstract. The following additional information was extracted from each included study: sample size, pathology, demographic information, support level, support type, methods, results, design, time since diagnosis, other information deemed relevant, and potential biasing factors. Patient responses and opinions were read by two independent reviewers (ML and MB) to identify common themes and subthemes, which were tabulated.

### Risk of bias (RoB)

**Fig. 2:**
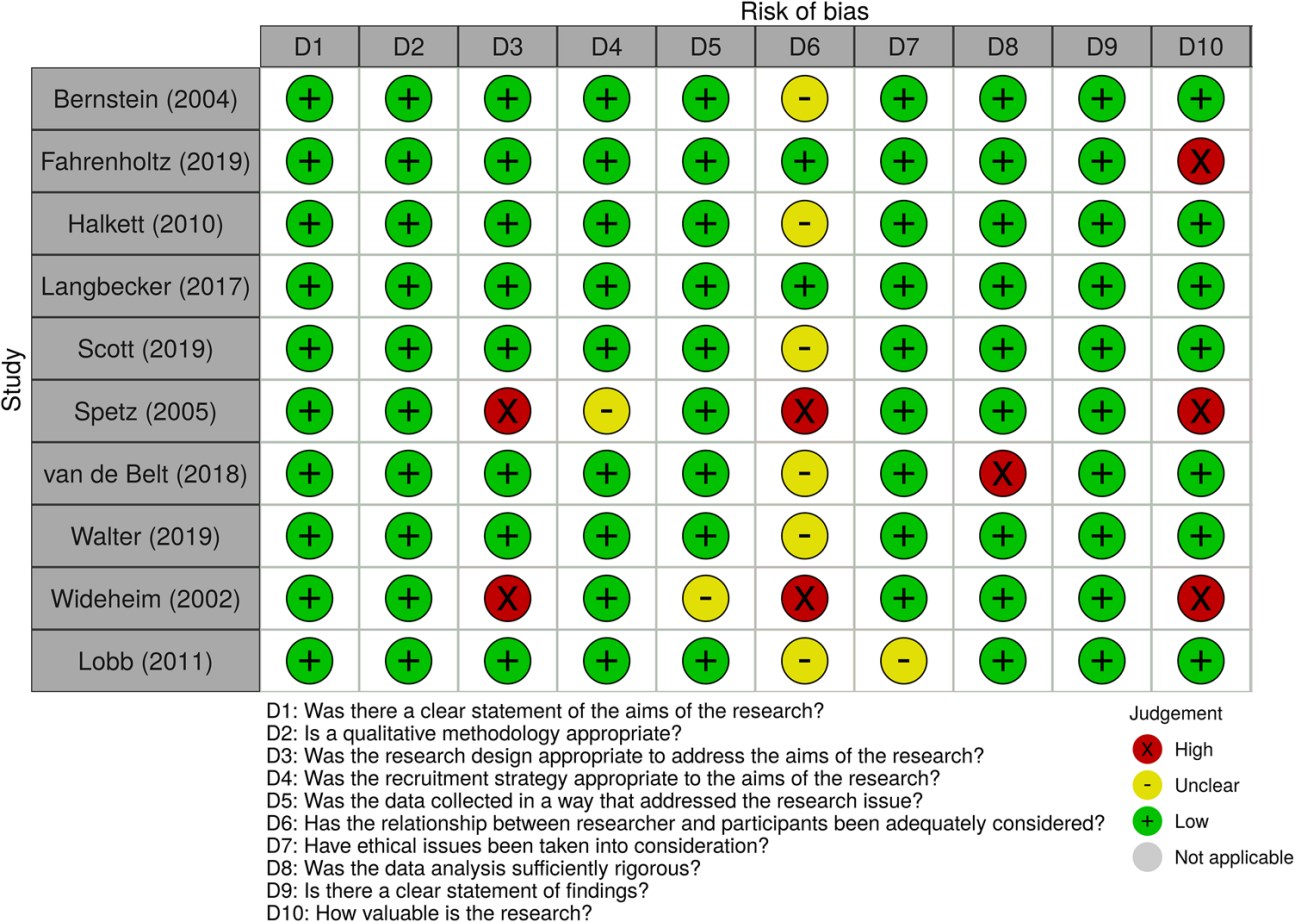
Risk of bias (RoB) in individual qualitative studies, based on the Critical Appraisal Skills Programme (CASP) Qualitative Checklist. Domains 1–10 were evaluated for each study and RoB was judged as high (X), unclear (−), or low (+)

Two independent reviewers (ML and JE) evaluated RoB. The cohort studies section of Newcastle–Ottawa Scale (NOS) [31] was used for quantitative, non-randomised studies (Table 1) which were allocated a score (0–9); those with a score equal or greater to six were judged as high-quality [32]. The Critical Appraisal Skills Program (CASP) Qualitative Studies Checklist [33] was used for qualitative studies (Figs. 2 and Supplementary Fig. S1).

**Table 1:** Risk of bias (RoB) for quantitative, non-randomised studies evaluated with the Newcastle–Ottawa Scale (NOS)

Study	Selection (0–3)	Comparability (0–2)	Outcome (0–3)
Diaz (2009)	★	★★	0
Langbecker (2016)	★★★★	★	★
Lucchiari (2010)	0	★★	★
Philip (2018)	★★★	★	★★

A greater number of stars indicates greater study quality for each domain (selection, comparability, outcome)

## Results

**Fig. 3:**
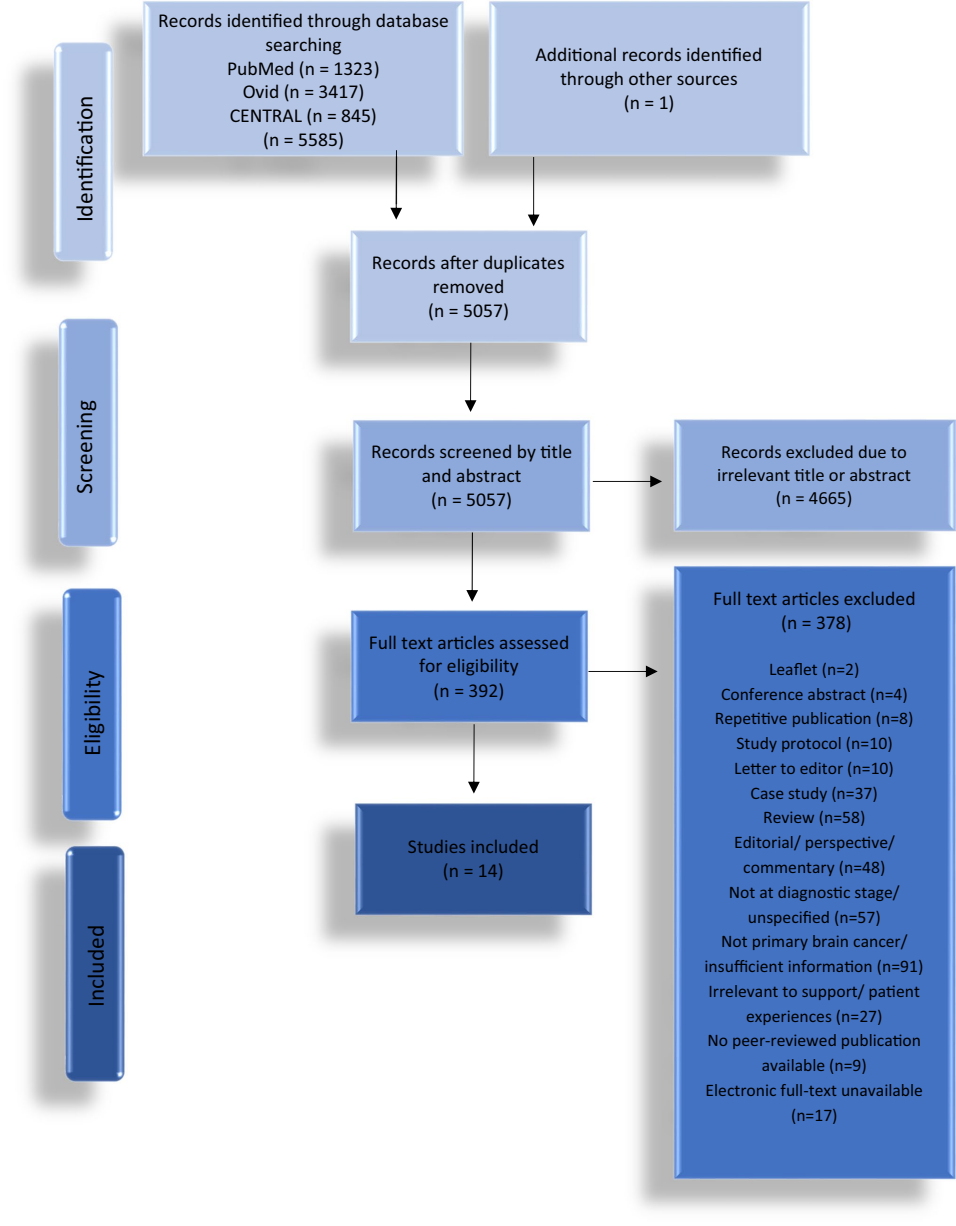
PRISMA flow diagram

The search, performed on November 10th 2020, resulted in 1323 articles on PubMed, 3417 on Embase (via Ovid) and 845 on CENTRAL. One article was found via reference-list search. Following deduplication, 5057 articles remained and were screened based on title and/or abstract. A total of 392 articles were retrieved for full-text review, with 14 deemed eligible. Due to the heterogeneity of study designs and their predominantly qualitative nature, a qualitative analysis was applied in this systematic review. The process is summarised in Fig. 3.

### Characteristics of included studies

Five studies were conducted in Australia, one in North America (Canada), and eight in Europe (United Kingdom, Sweden, Denmark, Netherlands, Italy, and Spain). Four studies were surveys, eight were semi-structured interviews, and two were conversational interviews. Included studies were categorised according to the assessed component of support: (a) format and level of information provided to patients was discussed in seven studies, (b) ongoing communication with patients in eight, and (c) patient participation in treatment also in eight studies. A summary of individual studies is provided (see Supplementary Table 1).

Patient age, sex, and pathology were reported across all studies, with additional demographics reported based on the experimental question. Patients across 14 included studies were diagnosed with malignant glioma. Histopathology and WHO grade were reported in seven studies and included: astrocytoma grade III to IV (*N* = 18) [34–36], anaplastic astrocytoma grade III (*N* = 29) [36, 37], anaplastic gemistocytic astrocytoma grade III (*N* = 1) [35], glioblastoma grade IV (*N* = 157) [5, 14, 35–38]. Sample sizes ranged from 5 to 39 and 26 to 84 participants for qualitative and quantitative studies, respectively. Sample size was driven by data saturation in qualitative studies, and statistical power in quantitative analyses.

Findings are discussed according to support type: (a) format and level of information provided to patients, (b) ongoing communication with patients, and (c) patient participation in treatment, and according to the macro- and micro-levels for each support type. Factors that increased or decreased perceived support were tabulated (Table 2). RoB was high for two quantitative studies [37, 39], moderate for two qualitative studies [34, 40], and low for the remaining ten studies [5, 14, 15, 35, 36, 38, 41–44].

**Table 2:** Guidelines based on evidence from extracted data indicating factors that increase or decrease patients’ perceptions of support

Factor effect	Support level	Factor	*N/n	Study First Author
Increased perceived support	*Macro*	Assigned care-coordinator to help with aspects of care (link between patient and healthcare system)	40/24	Langbecker [14]
			32/32	Philip [36]
			32/16	Spetz [34]
		Resource folder with general and personalised information (illness, symptoms, treatment, contact details)	40/24	Langbecker [14]
			32/32	Philip [36]
		Tumour visualisation with personalised 3D models	11/10	Van de Belt [43]
		Brief process of clinical investigation before diagnosis	5/5	Fahrenholtz [44]
		Visits to radiotherapy department and information about procedure and treatment side-effects	8/3	Wideheim [40]
	*Micro*	Physician willingness to answer questions	30/25	Bernstein [41]
			19/19	Halkett [42]
		Assessment of individual information needs/ preferences (e.g., medium, detail, framing, timing, etc.)	19/19	Halkett [42]
			40/19	Lobb [35]
		Physician’s encouragement to expand on symptoms/ observed changes	39/29	Walter [38]
		Physician booking/ encouraging patient to book next appointment	39/29	Walter [38]
		Healthcare team discussing potential of postoperative complications	30/25	Bernstein [41]
		Physician reputation (online/ by other professionals, patients)	30/25	Bernstein [41]
		Friendly, honest, direct physician	30/25	Bernstein [41]
		Ensuring the highest quality of care, despite terminal nature of disease	40/19	Lobb [35]
		Positively phrased prognosis (e.g., “you have six months left, not two, but six”)	40/19	Lobb [35]
Decreased perceived support	*Macro*	Too much or too little involvement in treatment decision-making	84/84	Lucchiari [37]
			19/19	Halkett [42]
		Long waiting time for an appointment, in-between appointments, or slow referrals	39/39	Scott [5]
			39/29	Walter [38]
		Poor continuity of care (e.g., patient has to repeat medical history to each new physician)	39/39	Scott [5]
			19/19	Halkett [42]
		Unavailability of preferred physician	39/39	Scott [5]
		Short appointments	39/29	Walter [38]
		Barriers to accessing professional support services (e.g., cost, complex paperwork)	19/10	Langbecker [15]
		Gaps between diagnosis and operation (> 3 weeks)	8/3	Wideheim [40]
	*Micro*	Disagreement between patient and physician on what comprises a symptom	39/29	Walter [38]
		Before diagnosis; physician not eager to investigate cause of symptoms further	39/29	Walter [38]
		Negatively phrased prognosis (e.g., “said there was no hope”)	40/19	Lobb [35]
		Delivering diagnosis and prognosis right after surgery	19/19	Halkett [42]
		Poor awareness of cancer-related symptoms that can be improved with professional help	19/10	Langbecker [15]
		Lack of information about the operation process	8/3	Wideheim [40]
		Receiving broad information (e.g., quantify slow progression)	8/3	Wideheim [40]
		Use of terminology during consultation (e.g., ‘glioma’, ‘malignant’, etc.)	8/3	Wideheim [40]

Factors are listed in descending order, according to the number of participants with malignant brain tumours within each level (Macro/Micro). *N/n = Total number of participants/ number of participants with malignant brain tumours

### Format and level of information provided to patients

Five studies discussed the provision of information on the macro-level [14, 35, 36, 39, 42] and five on the micro-level [35, 39–42].

#### Macro-level

Providing personalised resource folders immediately after diagnosis, significantly reduced (*P* = 0.001) 32 patients’ information needs after completion of radiotherapy [36]. However, mere awareness of the healthcare professionals (e.g., physiotherapist) patients can access, did not significantly reduce their information needs [14], indicating that professional guidance to utilise services is critical.

Cancer-related problems and indifference to seek additional information can create information gaps [35]. Information mediums need to cater to patients’ cancer-related deficits. Data from 19 patient interviews indicate a preference for written material in those unable to retain verbal information due to cancer-related memory problems [42]. Further, evaluation of 26 patients showed that those younger than 65 years tended to request additional information about their condition, contrary to older participants [39].

#### Micro-level

The treating physician is key in evaluating patients’ preferences relative to information parameters, including: (i) preferred level of detail [39], (ii) information format or medium (e.g., verbal or written) [42], (iii) timing [42], and (iv) framing (e.g., positively framed or neutral) [35]. Semi-structured interviews of 19 patients showed that poor understanding of medical terms (e.g., “glioma”), inadequate information about operation details and dissatisfaction with the use of broad terms (e.g., “slow tumour progression”), exacerbated their anxiety by impairing their comprehension [40]. Conversely, in semi-structured interviews of two independent patient cohorts, physicians’ willingness to answer questions improved perceived quality of care [41, 42].

### Ongoing communication with patients

Communication with patients was addressed in nine studies, with three referring to the macro-level [14, 34, 36] and six to the micro-level [15, 35, 38, 41, 43, 44].

#### Macro-level

At the macro-level, specialist nurses (SN) or care coordinators [14, 34, 36], provided practical advice, support, and served as first points of contact [34]. However, even when utilised at the diagnostic stage, these services failed to significantly reduce (*P* = 0.557) patients’ supportive needs by completion of radiotherapy [36].

#### Micro-level

Reluctance to utilise support services despite good self-reported awareness may be attributable to lack of knowledge on whether experienced symptoms were cancer-related [15], emphasising the value of physician communication. The time-consuming paperwork, additional financial burden, or inconvenient location further discouraged people from accessing professional support services [15]. Indeed, accessibility and awareness of potential benefits determines utilisation of services according to patient reports [44].

Five studies explored patient-physician communication and perceived support [35, 38, 41, 43, 44]. Honesty of healthcare staff increased perceptions of trust, despite the possibility of intraoperative complications, in a sample of 25 patients [41]. Likewise, maintaining realistic hope (e.g., “you have six months left, not two, but six”), denoted care would not be downgraded despite an incurable disease [35]. In a cohort of 19 patients and 21 caregivers only two had a positive experience with physician communication, with lack of empathy and compassion reported as critiques [35].

Patients valued physicians’ encouragement to discuss any observed changes and reassurance on the validity of symptoms, particularly when uncertain of which symptoms were worth disclosing [38]. Indifference of physicians to investigate symptoms further created dissatisfaction, leading patients to downplay their symptoms [38], whereas a friendly physician–patient relationship created strong rapport [44]. Physical aids to visualise the tumour and adjacent areas (e.g., three-dimensional, 3D, printed brains) can improve physician–patient communication without altering physicians’ communication style [43].

### Patient participation in treatment

Patient participation in treatment was examined in eight studies and was only apprehended on the macro-level [5, 37, 38, 40–44].

#### Macro-level

Prognostic uncertainty and treatment side-effects were discussed as factors associated with  increased anxiety in 19 patient interviews [42]. Thus, opportunities to receive accurate information about treatment procedures, such as through visits to the radiotherapy department, can be helpful [40].

Ten patients had personalised 3D brain models printed, which were used during consultations, facilitating treatment decisions with three additional reported benefits: (a) improved perceived comprehension and recall of their condition and surgical complications, (b) better coping, and (c) improved perceived physician–patient communication [43]. Nevertheless, personal preferences with regards to the degree of patient participation and 3D model visualisation of their condition were not unanimously positive with four patients reporting a negative effect [43]. Indeed, in quantitative assessment of the information management needs and treatment decisions of 84 patients, only 27 (32.14%) were satisfied with the received information and subsequently their treatment decision, while 29 (34.5%) were dissatisfied [37].

Participation in treatment extends to physician choice [5, 41] and physician reputation can increase patients’ trust and confidence [41]. However, qualitative reports of two independent samples of 39 and 29 patients, respectively, indicated long waiting times for appointments with patient-chosen physicians, preventing access to timely care [5, 38]. When physician choice was not feasible, patients reported poor continuity of care [5, 42]. Consequently, patients access emergency care [5, 38], with 27 patients reporting doing so, out of a cohort of 39 [5].

Likewise, slow inter-specialist referrals and brief consultations discouraged people from actively engaging with their treatment in a cohort of 29 patients [38]. Similarly, while extensive gaps between imaging and surgery intensified anxiety [40], brief clinical investigations before surgery promoted coping [44].

## Discussion

Existing literature, including four previous reviews on support systems of brain tumour patients, have only focused on palliative care [26, 28], interventions [45], telemedicine [46], non-medical therapies [28], and caregivers [28]. The current systematic review examined patient support services in the acute diagnostic period based on qualitative analysis of 14 studies, classified into three support domains: (1) format and level of information provided to patients [14, 35, 36, 39–42], (2) ongoing communication with patients [14, 15, 34, 35, 38, 41, 43, 44, 47] and (3) patient participation in treatment [5, 38, 40–44]. Four studies were cross-sectional and ten were descriptive. RoB was evaluated using the NOS and the CASP Qualitative Studies Checklist and was low in ten studies [5, 14, 15, 35, 36, 38, 41–44], moderate in two [34, 40], and high in two [37, 39]. Qualitative synthesis indicated that individualised care increased patients’ perceptions of support, contrary to poor patient-physician communication and complexity of the healthcare system. Extracted data were compiled as a list of guidelines (Table 2) that can apply to different healthcare systems with several factors emerging regarding the unmet supportive needs of brain tumour patients.

### Format and level of information provided to patients

At the macro-level, minimum information requirements include data about the condition, symptoms, and treatment options [14, 36] and our findings consistently reveal the need for individualised information [35, 39, 42]. Ideally, preferences and needs should be identified directly during physician–patient communication [37, 39, 42]. Evidence indicates that before a brain tumour diagnosis 24.9% of patients present with mental status changes [48], and therefore delivery of information should be adapted to individual cancer-related difficulties (e.g., memory impairment) [42]. Common demographic parameters such as age [39] or marital status can be influencing factors and hence should also be assessed, since information needs are significantly higher for cancer patients living alone, than with a partner (*P* = 0.02) [49]. Accommodating for patients’ circumstances when delivering information can improve comprehension and retention.

### Ongoing communication with patients

SN services promote patient-healthcare system communication [34, 36]. Qualitative evidence demonstrates these services assist with both medical and non-medical cancer-related difficulties [34], yet fail to significantly reduce patients’ needs quantitatively [36]. However, these data should be interpreted with caution as further research is needed. The wide scope of support offered by SN services is unlikely to be captured by a single quantitative measure.

Data showed patients have good awareness of available support services, yet are unable to distinguish purely psychological from tumour-related symptoms [15]. Therefore, patients may decline professional help due to false assumptions regarding which symptoms can or cannot be improved [15]. Physicians can refer patients to appropriate services, with future research considering interventions to promote engagement, considering the high prevalence of psychological disorders among brain cancer patients [50]. On the macro-level, question-prompt lists have been shown to increase patient participation, encouraging significantly more targeted questions during consultations (*P* = 0.048) compared to controls [51].

Satisfaction with physicians’ communication style can increase engagement [38] and confidence in the competency of the healthcare team [41]. Indeed, physicians’ scores on empathy and attentiveness significantly (*P* < 0.01) correlated with patient satisfaction in a cohort of 500 oncology patients [52]. Alternatively, use of aids (e.g., 3D-printed brains) can facilitate communication without requiring adaptations of the physician’s communication style [43].

### Patient participation in treatment

Quantitative data illustrated patients’ dissatisfaction with their degree of involvement in therapeutic decisions [37]. Consistently, a multicentre study on 480 breast cancer patients, showed 44% preferred physician-directed decisions, while 45% favoured a shared-decision approach [53]. Consultation length (median time 30 min) was a significant predictor (*P* = 0.02) of satisfaction, demonstrating the importance of physician communication in all aspects of care [53]. In malignant brain tumour cohorts, the uncertain nature of the disease reportedly increased treatment decision-making burden [42], suggesting that individual diagnoses may also be a critical variable. Further, higher patient age concurred with preferences for decreased involvement in care [54], with ethnicity and language presenting additional barriers to patient involvement [55].

## Reforming of services

This systematic review is based on studies from eight different healthcare systems. Despite the underlying diversity, extracted data (Table 2) may apply to various services treating patients with malignant brain tumours. Different healthcare systems can select and adapt factors accordingly.

According to the NHS Long Term Plan and the NHS Model of Personalised Care, cancer patients should receive holistic needs assessment, personalised information, and access to support services [56]. Based on our findings, the following four-step plan can be further implemented to maximise support. First, sociodemographic information could identify patients needing specific support services or adjustments, such as financial aid. Second, routine patient satisfaction surveys at the acute diagnostic period could identify gaps in patient-physician communication, which can then be addressed in targeted communication skills training offered to healthcare staff. Lastly, voluntary seminars on the range of available support services could be offered to patients.

## Limitations

Sample sizes were small across all included studies in keeping with the low incidence of malignant brain tumours and investigation of an overlooked subject (i.e., patient support systems) [57]. Our stringent criteria concerning the short period between diagnosis and assessment limited the number of available studies, although minimised survivor bias. Further, only four quantitative studies were eligible, restricting investigation of our topic using numerical assessments. Although qualitative analyses allowed for the extraction of data rich in patient insight, the exact number of participants reporting a particular experience was not routinely reported. Further, qualitative data are often subject to researcher bias; to overcome this, direct quotes were analysed where possible. Recall bias due to cancer-related memory impairments was considered likely in two retrospective studies [35, 42]. Lastly, analysed data reflect the experiences of malignant brain tumour patients, which might differ from other cohorts.

## Conclusion

Establishing a support system for brain cancer patients within the first few months of diagnosis is critical in maximising care quality. This systematic review analyses current support systems while providing: (i) an evidence-based list of factors needing improvement and (ii) a four-step recommendation plan for healthcare services. The evidence-based factors aim to guide revisions of existing support systems for patients with malignant brain tumours.

## Supplementary Information

Below is the link to the electronic supplementary material.

Supplementary file1 (PDF 739 kb)

## Data Availability

All data generated or analysed during this study are included in this published article and its supplementary information files.

## References

[1] Chambers SK, Grassi L, Hyde MK (2015). Integrating psychosocial care into neuro-oncology: challenges and strategies. Front Oncol.

[2] Pascoe SW, Neal RD, Allgar VL (2004). Psychosocial care for cancer patients in primary care? Recognition of opportunities for cancer care. Fam Pract.

[3] Taphoorn MJB, Claassens L, Aaronson NK (2010). An international validation study of the EORTC brain cancer module (EORTC QLQ-BN20) for assessing health-related quality of life and symptoms in brain cancer patients. Eur J Cancer.

[4] Meyers CA, Hess KR (2003). Multifaceted end points in brain tumor clinical trials: cognitive deterioration precedes MRI progression. Neuro Oncol.

[5] Scott SE, Penfold C, Saji S (2019). ‘It was nothing that you would think was anything’: qualitative analysis of appraisal and help seeking preceding brain cancer diagnosis. PLoS ONE.

[6] UK CR Brain, other CNS and intracranial tumours statistics. https://www.cancerresearchuk.org/health-professional/cancer-statistics/statistics-by-cancer-type/brain-other-cns-and-intracranial-tumours. Accessed 24 Nov 2020

[7] Renovanz M, Hickmann AK, Coburger J (2018). Assessing psychological and supportive care needs in glioma patients – feasibility study on the use of the Supportive Care Needs Survey Short Form (SCNS-SF34-G) and the Supportive Care Needs Survey Screening Tool (SCNS-ST9) in clinical practice. Eur J Cancer Care.

[8] Renovanz M, Maurer D, Lahr H (2018). Supportive care needs in glioma patients and their caregivers in clinical practice: results of a multicenter cross-sectional study. Front Neurol.

[9] Kohlmann K, Janko M, Ringel F, Renovanz M (2019). Self-efficacy for coping with cancer in glioma patients measured by the cancer behavior inventory brief version. Psychooncology.

[10] Sterckx W, Coolbrandt A, Dierckx de Casterlé B (2013). The impact of a high-grade glioma on everyday life: a systematic review from the patient’s and caregiver’s perspective. Eur J Oncol Nurs.

[11] Madhusoodanan S, Ting MB, Farah T, Ugur U (2015). Psychiatric aspects of brain tumors: a review. World J Psychiatry.

[12] Nyamathi A (1989). Comprehensive health seeking and coping paradigm. J Adv Nurs.

[13] Moorey S, Frampton M, Greer S (2003). The cancer coping questionnaire: a self-rating scale for measuring the impact of adjuvant psychological therapy on coping behaviour. Psychooncology.

[14] Langbecker D, Yates P (2016). Primary brain tumor patients’ supportive care needs and multidisciplinary rehabilitation, community and psychosocial support services: awareness, referral and utilization. J Neurooncol.

[15] Langbecker D, Ekberg S, Yates P (2017). Don’t need help, don’t want help, can’t get help: How patients with brain tumors account for not using rehabilitation, psychosocial and community services. Patient Educ Couns.

[16] Andersen BL, Farrar WB, Golden-Kreutz D (2007). Distress reduction from a psychological intervention contributes to improved health for cancer patients. Brain Behav Immun.

[17] Jenkinson C, Coulter A, Bruster S (2002). The picker patient experience questionnaire: development and validation using data from in-patient surveys in five countries. Int J Qual Health Care.

[18] Bonevski B, Sanson-Fisher R, Girgis A (2000). Evaluation of an instrument to assess the needs of patients with cancer. Cancer.

[19] Girgis A, Stojanovski E, Boyes A (2012). The next generation of the supportive care needs survey: a brief screening tool for administration in the clinical oncology setting. Psychooncology.

[20] National Clinical Guideline Centre (UK) (2012) Patient experience in adult NHS services: improving the experience of care for people using adult NHS services: patient experience in generic terms. Royal College of Physicians (UK) 23285499

[21] Merluzzi TV, Philip EJ, Heitzmann Ruhf CA (2018). Self-efficacy for coping with cancer: revision of the cancer behavior inventory (version 3.0). Psychol Assess.

[22] Blanchard CG, Labrecque MS, Ruckdeschel JC, Blanchard EB (1990). Physician behaviors, patient perceptions, and patient characteristics as predictors of satisfaction of hospitalized adult cancer patients. Cancer.

[23] National Collaborating Centre for Cancer (UK) (2015) Suspected cancer: recognition and referral. National Institute for Health and Care Excellence (NICE) 26180880

[24] Wiggers JH, Donovan KO, Redman S, Sanson-Fisher RW (1990). Cancer patient satisfaction with care. Cancer.

[25] Cavers D, Hacking B, Erridge SC (2013). Adjustment and support needs of glioma patients and their relatives: serial interviews. Psychooncology.

[26] Crooms RC, Goldstein NE, Diamond EL, Vickrey BG (2020). Palliative care in high-grade glioma: a review. Brain Sci.

[27] Song K, Amatya B, Voutier C, Khan F (2016). Advance care planning in patients with primary malignant brain tumors: a systematic review. Front Oncol.

[28] Ford E, Catt S, Chalmers A, Fallowfield L (2012). Systematic review of supportive care needs in patients with primary malignant brain tumors. Neuro Oncol.

[29] Shamseer L, Moher D, Clarke M (2015). Preferred reporting items for systematic review and meta-analysis protocols (PRISMA-P) 2015: elaboration and explanation. BMJ.

[30] Moher D, Shamseer L, Clarke M (2015). Preferred reporting items for systematic review and meta-analysis protocols (PRISMA-P) 2015 statement. Syst Rev.

[31] Stang A (2010). Critical evaluation of the Newcastle-Ottawa scale for the assessment of the quality of nonrandomized studies in meta-analyses. Eur J Epidemiol.

[32] Farsad-Naeimi A, Asjodi F, Omidian M (2020). Sugar consumption, sugar sweetened beverages and attention deficit hyperactivity disorder: a systematic review and meta-analysis. Complement Ther Med.

[33] Jaeschke R, Guyatt GH, Sackett DL (1994). Users’ guides to the medical literature. III. How to use an article about a diagnostic test. B. What are the results and will they help me in caring for my patients? The evidence-based medicine working group. JAMA.

[34] Spetz A, Henriksson R, Bergenheim AT, Salander P (2005). A specialist nurse-function in neurooncology: a qualitative study of possibilities, limitations, and pitfalls. Palliat Support Care.

[35] Lobb EA, Halkett GKB, Nowak AK (2011). Patient and caregiver perceptions of communication of prognosis in high grade glioma. J Neurooncol.

[36] Philip J, Collins A, Staker J, Murphy M (2019). I-CoPE: a pilot study of structured supportive care delivery to people with newly diagnosed high-grade glioma and their carers. Neuro-Oncol Pract.

[37] Lucchiari C, Botturi A, Pravettoni G (2010). The impact of decision models on self-perceived quality of life: a study on brain cancer patients. Ecancermedicalscience.

[38] Walter FM, Penfold C, Joannides A (2019). Missed opportunities for diagnosing brain tumours in primary care: a qualitative study of patient experiences. Br J Gen Pract.

[39] Díaz JL, Barreto P, Gallego JM (2009). Proper information during the surgical decision-making process lowers the anxiety of patients with high-grade gliomas. Acta Neurochir (Wien).

[40] Wideheim AK, Edvardsson T, Påhlson A, Ahlström G (2002). A family’s perspective on living with a highly malignant brain tumor. Cancer Nurs.

[41] Bernstein M, Potvin D, Martin DK (2004). A qualitative study of attitudes toward error in patients facing brain tumour surgery. Can J Neurol Sci.

[42] Halkett GKB, Lobb EA, Oldham L, Nowak AK (2010). The information and support needs of patients diagnosed with high grade glioma. Patient Educ Couns.

[43] van de Belt TH, Nijmeijer H, Grim D (2018). Patient-specific actual-size three-dimensional printed models for patient education in glioma treatment: first experiences. World Neurosurg.

[44] Fahrenholtz ML, Hansen A, Søgaard K, Andersen LN (2019). Finding “the inner drive” for a rehabilitation process: a small-scale qualitative investigation among male patients with primary glioma. BMJ Open.

[45] Piil K, Juhler M, Jakobsen J, Jarden M (2016). Controlled rehabilitative and supportive care intervention trials in patients with high-grade gliomas and their caregivers: a systematic review. BMJ Support Palliat Care.

[46] Ownsworth T, Chan RJ, Jones S (2020). Use of telehealth platforms for delivering supportive care to adults with primary brain tumors and their family caregivers: a systematic review. Psychooncology.

[47] Rossit S, Benwell CSY, Szymanek L (2019). Efficacy of home-based visuomotor feedback training in stroke patients with chronic hemispatial neglect. Neuropsychol Rehabil.

[48] Comelli I, Lippi G, Campana V (2017). Clinical presentation and epidemiology of brain tumors firstly diagnosed in adults in the emergency department: a 10-year, single center retrospective study. Ann Transl Med.

[49] Voogt E, Van Leeuwen AF, Visser AP (2005). Information needs of patients with incurable cancer. Support Care Cancer.

[50] Wellisch DK, Kaleita TA, Freeman D (2002). Predicting major depression in brain tumor patients. Psychooncology.

[51] Brown R, Butow PN, Boyer MJ, Tattersall MHN (1999). Promoting patient participation in the cancer consultation: evaluation of a prompt sheet and coaching in question-asking. Br J Cancer.

[52] Zachariae R, Pedersen CG, Jensen AB (2003). Association of perceived physician communication style with patient satisfaction, distress, cancer-related self-efficacy, and perceived control over the disease. Br J Cancer.

[53] Hitz F, Ribi K, Li Q (2013). Predictors of satisfaction with treatment decision, decision-making preferences, and main treatment goals in patients with advanced cancer. Support Care Cancer.

[54] Lam W, Fielding R, Chan M (2003). Participation and satisfaction with surgical treatment decision-making in breast cancer among Chinese women. Breast Cancer Res Treat.

[55] López ME, Kaplan CP, Nápoles AM (2014). Satisfaction with treatment decision-making and treatment regret among Latinas and non-Latina whites with DCIS. Patient Educ Couns.

[56] NHS (2019) The NHS long term plan. https://www.longtermplan.nhs.uk/

[57] Bondy ML, Scheurer ME, Malmer B (2008). Brain tumor epidemiology: consensus from the brain tumor epidemiology consortium. Cancer.

