# Supplementary material for: Reforming support systems of newly diagnosed brain cancer patients: a systematic review
Source: J Neurooncol. 2021 Nov 26;156(1):61–71. doi: 10.1007/s11060-021-03895-4 (PMC8714629; doi:10.1007/s11060-021-03895-4)
Supplement: Supplementary file 1 — Supplementary file1 (PDF 739 kb) [file 11060_2021_3895_MOESM1_ESM.pdf]

Reforming support systems of newly diagnosed brain cancer patients: A systematic review

Journal of Neuro-Oncology

Maria Loizidou, Viktoria Sefcikova, Justyna O. Ekert, Matan Bone, George Samandouras

Corresponding Author: Maria Loizidou
UCL Queen Square Institute of Neurology, University College London, Queen Square, London, United Kingdom
Email: maria.loizidou.20@ucl.ac.uk

Supplementary data

Search strategies used for PubMed, Embase (Classic + Embase) via Ovid and CENTRAL.

1. Search strategy for PubMed:

(A)

(1) professional-patient relations [MeSH]

(2) health education [MeSH] OR health education [Text Word]

(3) patient education as topic [MeSH] OR patient education [Text Word]

(4) self efficacy [MeSH] OR self efficacy [Text Word]

(5) self-help devices [MeSH] OR self-help device* [Text Word]

(6) Community health* [Text Word]

(7) telemedicine [MeSH] OR telemedicine [Text Word]

(8) psychosocial support systems [MeSH] OR psychosocial support [Text Word]

(9) patient support [Text Word]

(10) patient communication [Text Word]

Search: (professional-patient relations [MeSH]) OR (health education [MeSH] OR health education [Text Word]) OR (patient education as topic [MeSH] OR patient education [Text Word]) OR (self efficacy [MeSH] OR self efficacy [Text Word]) OR (self-help devices [MeSH] OR self-help device* [Text Word]) OR (community health* [Text Word]) OR (telemedicine [MeSH] OR telemedicine [Text Word]) OR (psychosocial support systems [MeSH] OR psychosocial support [Text Word]) OR (patient support [Text Word]) OR (patient communication [Text Word])

(B)

(1) glioma [MeSH] OR glioma [Text Word]

(2) brain tumor* [Text Word]

(3) brain tumour* [Text Word]

(4) neurosurgery [MeSH] OR neurosurgery [Text Word]

(5) brain neoplasms [MeSH] OR brain neoplasms [Text Word]

Search: (glioma [MeSH] OR glioma [Text Word]) OR (brain tumor* [Text Word]) OR (brain tumour* [Text Word]) OR (neurosurgery [MeSH] OR neurosurgery [Text Word]) OR (brain neoplasms [MeSH] OR brain neoplasms [Text Word])

Query box: ((professional-patient relations [MeSH] OR professional-patient relations [Text Word]) OR (health education [MeSH] OR health education [Text Word]) OR (patient education as topic [MeSH] OR patient education [Text Word]) OR (self efficacy [MeSH] OR self efficacy [Text Word]) OR (self-help devices [MeSH] OR self-help device* [Text Word]) OR (telemedicine [MeSH] OR telemedicine [Text Word]) OR (psychosocial support systems [MeSH] OR psychosocial support systems [Text Word]) OR (patient support [Text Word]) OR (patient communication [Text Word])) AND ((glioma [MeSH] OR glioma [Text Word]) OR (brain tumor [Text Word]) OR (brain tumour [Text Word]) OR (neurosurgery [MeSH] OR neurosurgery [Text Word]) OR (brain neoplasms [MeSH] OR brain neoplasms [Text Word]))

((professional-patient relations [MeSH]) OR (health education [MeSH] OR health education [Text Word]) OR (patient education as topic [MeSH] OR patient education [Text Word]) OR (self efficacy [MeSH] OR self efficacy [Text Word]) OR (self-help devices [MeSH] OR self-help device* [Text Word]) OR (community health* [Text Word]) OR (telemedicine [MeSH] OR telemedicine [Text Word]) OR (psychosocial support systems [MeSH] OR psychosocial support [Text Word]) OR (patient support [Text Word]) OR (patient communication [Text Word])) AND (((((oligoastrocytoma[MeSH Terms]) OR (oligoastrocytoma[Text Word])) OR ((meningioma[MeSH Terms]) OR (meningioma[Text Word]))) OR ((oligodendroglioma[MeSH Terms]) OR (oligodendroglioma[Text Word]))) OR ((astrocytoma[MeSH Terms]) OR (astrocytoma[Text Word])))

2. Search strategy for EMBASE (Classic + Embase) via Ovid

(1) exp glioma/
(2) glioma*.mp.
(3) exp neurosurgery/
(4) neurosurgery.mp.
(5) exp brain neoplasm/

(6) brain neoplasm.mp.

(7) exp brain tumor/

(8) exp brain tumour/

(9) brain tumor*.mp

(10) brain tumour*.mp
(11) 1 or 2 or 3 or 4 or 5 or 6 or 7 or 8 or 9 or 10
(12) exp professional patient relations/

(13) exp health education/

(14) health education.mp.

(15) exp patient education as topic/

(16) patient education.mp.

(17) exp self efficacy/

(18) self efficacy.mp.

(19) exp self help devices/

(20) self help device*.mp.

(21) exp community health services/

(22) community health*.mp.

(23) exp telemedicine/

(24) telemedicine.mp.

(25) exp psychosocial support system/

(26) psychosocial support.mp.

(27) patient commun*.mp.

(28) 12 or 13 or 14 or 15 or 16 or 17 or 18 or 19 or 20 or 21 or 22 or 23 or 24 or 25 or 26 or 27

(29) 11 and 28

3. Search strategy for CENTRAL:

(1) MeSH descriptor: [glioma] explode all trees

(2) Glioma*

(3) brain tumor*

(4) brain tumour*

(5) MeSH descriptor: [neurosurgery] explode all trees

(6) Neurosurgery

(7) MeSH descriptor: [brain neoplasms] explode all trees

(8) Brain neoplasm*

(9) #1 OR #2 OR #3 OR #4 OR #5 OR #6 OR #7 OR #8

(10) MeSH descriptor: [professional-patient relations] explode all trees

(11) MeSH descriptor: [patient education as topic] explode all trees

(12) Patient education

(13) MeSH descriptor: [health education] explode all trees

(14) Health education

(15) MeSH descriptor: [self efficacy] explode all trees

(16) Self efficacy

(17) MeSH descriptor: [self-help devices] explode all trees

(18) Self-help device*

(19) MeSH descriptor: [community health services] explode all trees

(20) Community health*

(21) MeSH descriptor: [telemedicine] explode all trees

(22) telemedicine

(23) MeSH descriptor: [psychosocial support systems] explode all trees

(24) Psychosocial support

(25) #10 OR #11 OR #12 OR #13 OR #14 OR #15 OR #16 OR #17 OR #18 OR #19 OR #20 OR #21 OR #22 OR #23 OR #24

(26) #9 AND #25

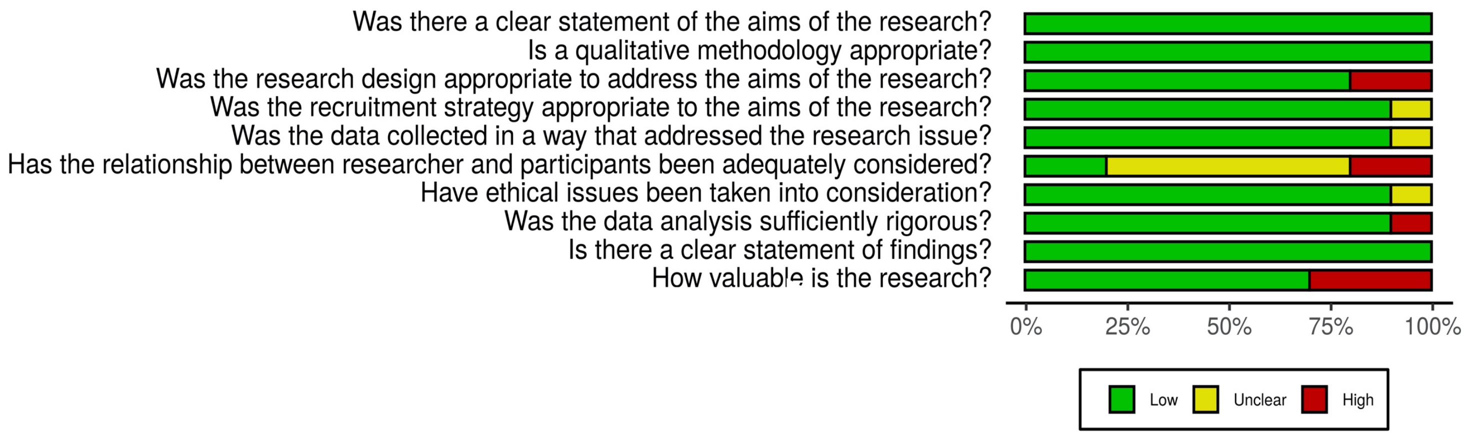

Supplementary Fig. 1 Summary plot of risk of bias for qualitative studies, evaluated with the Critical Appraisal Skills Program (CASP) Qualitative Studies Checklist.

Study first author	Design	N/n*	Time of testing	Support component	Level	Findings
Lucchiari [35]	Survey	84/84	< 3 months (follow-up 3 months later)	(c) Patient participation in treatment	Macro	Varying information needs and preferences for involvement in decision-making process. Patients satisfied with their involvement in their care and knowledge about their condition, had better emotional and social quality of life.
Scott [5]	Semi-structured interviews	39/39	4 weeks	(c) Patient participation in treatment	Macro	Dissatisfaction with complexity of healthcare system and inability to access medical help promptly.
Philip [36]	Survey	32/32	< 3 months	(a) Information provided to patients<br>(b) Communication with patients	Macro	Delivery of i-CoPE intervention at 3 critical transition points, when support needs are highest (after diagnosis, after hospital discharge, after completion of radiotherapy). Intervention included: assigning care-coordinator to each patient, providing a folder with personalised information (diagnosis, symptoms, etc) and regular screening for quality of life (physical, social, emotional, functional, brain-cancer-specific domains).
Walter [38]	Semi-structured interviews	39/29	< 4 weeks	(b) Communication with patients<br>(c) Patient participation in treatment	Macro	Patients valued physician prompts to expand on symptoms and eagerness to investigate them further. Complexity of the healthcare system (slow referrals, long waiting times for appointments) lowered patients' engagement with their physician.
Diaz [39]	Survey	26/26	preoperatively	(a) Information provided to patients	Both	Differing needs for information depending on personal preferences and age. Lower information needs in patients over 65 years old. Lower information needs, poorer comprehension and satisfaction with received information, correlated with increased anxiety.
Bernstein [41]	Semi-structured interviews	30/25	< 1 week after consultation	(a) Information provided to patients<br>(b) Communication with patients<br>(c) Patient participation in treatment	Both	For medical errors, trust in the medical professional was a mitigatory for concern. Physician experience, reputation, and presentation during consultation (honest, friendly, direct), increased patients' confidence in the operation, despite possibility of error.
Langbecker [14]	Survey	40/24	< 3 months (follow-up 3 months later)	(a) Information provided to patients<br>(b) Communication with patients	Macro	Utilisation of support services (for physical and psychological needs) was higher if patient had been referred to the service by their clinician. Patients reported decreased referral to psychological support services and higher unmet psychological needs.
Halkett [42]	Semi-structured interviews	19/19	<1 year	(a) Information provided to patients<br>(c) Patient participation in treatment	Both	Patients' information needs/preferences need to be assessed prior to providing information. Variability in information mediums, to ensure satisfaction and retaining of information, regardless of cancer-related symptoms (e.g. written information to patients with memory problems)
Lobb [35]	Semi-	40/19	Refers to	(a) Information provided to patients	Both	Patients appreciated feeling they would receive the best possible

	structured interviews		diagnostic period	(b) Communication with patients		care, despite the terminal nature of their condition. Physician communication was important in maintain patient hope or not (e.g. phrasing prognosis positively and not dwelling on the terminal nature of the tumour).
Spetz [34]	Conversational interviews	32/16	After diagnosis	(b) Communication with patients	Macro	Assigned specialised-nurse to patients after diagnosis. Patients utilised this contact for emotional support, medical advice, help with appointments, referral to other support services. Link between patient and healthcare system.
Van de Belt [43]	Semi-structured interviews	11/10	"after neurosurgical consultation"	(b) Communication with patients (c) Patient participation in treatment	Both	Use of personalised 3D models during consultation. Facilitated patients' understanding of location/size of tumour, treatment-associated risks and ability to ask more targeted questions.
Langbecker [15]	Semi-structured interviews	19/10	3 months (follow-up, 3 months later	(b) Communication with patients	Micro	Good awareness but lack of utilising support services, due to: desire to manage symptoms without help, prioritising medical treatment, complexity of paperwork, beliefs that symptoms cannot be improved.
Fahrenholtz [44]	Semi-structured interviews	5/5	"newly diagnosed"	(b) Communication with patients (c) Patient participation in treatment	Both	Information on importance of physical exercise in managing symptoms and functional ability. Access to physiotherapist/ physical exercise group therapy, shifted focus away from illness and increased perceived disease-related resilience.
Wideheim [40]	Conversational interviews	8/3	< 6 months	(a) Information provided to patients (c) Patient participation in treatment	Both	Need for more specific information (when referring to tumour progression, details about how the operation will be done). Poor understanding of medical terms (malignant, glioma, etc.). Patients engaged with opportunities to visit radiotherapy department prior to radiotherapy.

N/n* = Total number of participants/ number of participants with malignant brain tumours.

Supplementary Table 1 Summary of findings of included studies. Studies appear by decreasing number of participants with malignant brain tumours.
